# Efficient Sunlight-Induced Methylene Blue Removal over One-Dimensional Mesoporous Monoclinic BiVO_4_ Nanorods

**DOI:** 10.1155/2012/345247

**Published:** 2012-04-08

**Authors:** Linrui Hou, Long Yang, Jiaoyang Li, Jie Tan, Changzhou Yuan

**Affiliations:** Anhui Key Laboratory of Metal Materials and Processing, School of Materials Science and Engineering, Anhui University of Technology, Ma'anshan 243002, China

## Abstract

Sunlight-driven mesoporous BiVO_4_ nanorods with monoclinic structure have been successfully synthesized *via* a simple hydrothermal method. The as-prepared one-dimensional BiVO_4_ nanorods exhibited high specific surface area due to their unique mesoporous structure. The mesoporous BiVO_4_ nanorods possessed strong photoabsorption properties in the visible light region as well as the ultravisible region, and the band gap was estimated to be *ca.* 2.18 eV. The photocatalytic activities were evaluated by decolorization of methylene blue under sunlight irradiation. Photocatalytic tests demonstrated that the decolorization rate of as-prepared mesoporous BiVO_4_ nanorods was even up to 98.8% in 180 min, much better than that prepared by solid-state reaction (23.1%) and the commercial TiO_2_ (Degussa P25) (14.2%) under the same conditions, due to their higher specific surface area and appropriate band gap. Moreover, the unique BiVO_4_ nanorods exhibit high stability after five photocatalytic degradation recycles.

## 1. Introduction

Organic dyes in textile and industrial effluents have become the major environmental contaminants. Furthermore, many dyes are highly water-soluble, and some traditional treatment methods, such as activated carbon adsorption, flocculation, and biological treatment, do not work efficiently [1]. Thus it has become a challenging and indispensable topic of modern research how to efficiently solve this urgent environmental issue. Recently, photocatalysis technique has displayed its obvious advantage in comparison to the above treatment methods. The early study mainly focused on the TiO_2_-based photocatalysts, while TiO_2_ only responds to ultravisible (UV) irradiation, which occupies only* ca. *4% of the whole solar energy [2], which greatly hinders its further wider application. To utilize efficiently cheap solar energy, the development of sunlight-driven photocatalyst has become one of the most significant topics recently.

In the search of semiconductor materials with photocatalytic activity under sunlight irradiation, many efforts have been put on designing nontitania-based semiconductor photocatalysts including CdBi_2_O_4_ [3], Bi_3_O_4_Cl [4], and Bi_2_MoO_6_ [5], Ag_2_CrO_4_ [6], BiVO_4_ [7, 8], and Bi_2_MoO_6_ [9]. Those new ternary metal oxide semiconductors possess steep absorption edges in the visible-light region, which is obviously different from the structured spectrum of TiO_2_ [10]. In addition, compared to TiO_2_, the valence bands of the ternary metal oxide semiconductors consist of hybridizations of transition-metal orbitals, and this hybridization can increase the VB level, thus resulting in the narrow band gap. The narrower band gap of these photocatalysts will facilitate excitation of the electrons from the valence band (VB) to the conduction band (CB), which is beneficial to the photocatalytic oxidation reaction [11]. Moreover, for the photocatalytic degradation of dilute pollutants, the exposed surfaces of the photocatalysts will serve as centers of condensing substrates through a physical adsorption process, and the condensed substrates would be degraded by generated hydroxyl radical [12]. Therefore, a photocatalyst with large surface specific area (SSA) is also important for the efficient degradation of dilute pollutants.

As one of the nontitania-based visible-light-driven semiconductor photocatalysts, BiVO_4_ has attracted extensive interests for organic photocatalytic degradation due to its narrow band gap. BiVO_4_ crystallizes in three different polymorphs, that is, tetragonal zircon, monoclinic distorted scheelite, and tetragonal scheelite, in which monoclinic BiVO_4_ shows the highest photocatalytic activity [13, 14]. Thus, the synthesis of BiVO_4_ with monoclinic phase has become the focus of study today. Up to now, various methods have been used to synthesize BiVO_4_ with monoclinic phase, such as solid-state reaction method [15, 16], ultrasonic-assisted microemulsion method [17], aqueous process [18, 19], flame spray pyrolysis [20], hydrothermal method [21], and solvothermal route [22]. Among these synthetic methods, hydrothermal method is found to be facile and efficient for the preparation of BiVO_4_ with monoclinic phase [21].

In addition, the discharge of colored wastewater from industries has caused many environmental problems, and many dyestuffs cannot be degraded by a conventional method, such as biological treatment. Among all the nonbiodegradable stuffs, methylene blue (MB) is a commonly used dyestuff that has wider applications including coloring paper, temporary hair colorant, dyeing cottons, wools, and coating for paper stock. However, acute exposure to MB will cause health problems, such as increasing heart rate, vomiting, shock, Heinz body formation, cyanosis, and tissue necrosis in humans [23–25]. For this reason, it is imperative to develop the photocatalysts for the effective degradation of MB.

**Figure 1:**
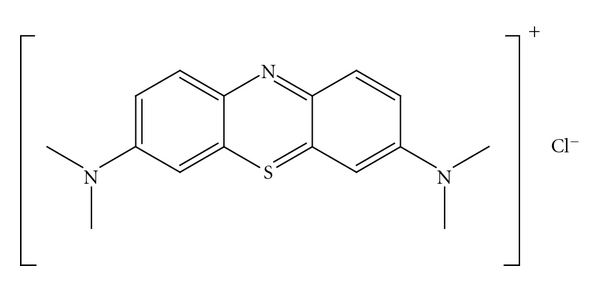
Structure of the methylene blue.

Based on the above overviews, in this paper, one-dimensional (1D) monoclinic BiVO_4_ nanorods with rich mesopores, to the best of our knowledge, were first synthesized by a facile hydrothermal method. The high sunlight-induced photocatalytic activity of BiVO_4_ nanorods was investigated based on MB as decomposed substance. For reference, the structure of MB is presented in Figure 1. Photocatalytic experiments demonstrated that MB was almost completely decomposed within only 180 min under sunlight irradiation, revealing their excellent photocatalytic activity under sunlight, which is of great significance to its practical applications.

## 2. Experimental

### 2.1. Preparation of Mesoporous BiVO_4_ Nanorods

All the reagents were of analytical purity and used without further purification. The typical synthesis of mesoporous BiVO_4_ nanorods was shown as follows: Bi(NO_3_)_3_·5H_2_O (3 mmol) and NH_4_VO_3_ (3 mmol) were firstly dissolved into 30 mL of dilute HNO_3_ aqueous solution (2 M), respectively, and then two nitric acid solutions of Bi(NO_3_)_3_·5H_2_O and NH_4_VO_3_ were mixed. Afterwards, the pH value of the mixed solution was adjusted to 6-7 by ethylenediamine. Finally, the precursor solution was transferred into a 100 mL Teflon-lined stainless-steel autoclave and then heated at 160°C for 5 h. Subsequently, it was cooled to room temperature, and the yellow precipitates were separated by filtration, washed with deionized water and absolute ethanol several times, and then dried at 60°C for 12 h in air. For comparison, another BiVO_4_ sample (denoted as SSR-BiVO_4_) was also prepared by a solid-state reaction according to the reference [26].

### 2.2. Characterization

The crystalline phase and image of the sample were determined by X-ray diffractometer (XRD, Max 18^XCE^, Japan) using a Cu Ka source (*λ* = 0.154056 nm), scanning electron microscopy (SEM, LEO 1403VP, Germany), and transmission electronic microscopy (TEM, Hitachi-600, Japan), respectively. To determine the band gap energy of the photocatalyst, UV-Vis diffuse reflectance spectrum was carried out, in the wavelength range of 200–800 nm, using a Hitachi U-3010 spectrophotometer. The pure powdered Al_2_O_3_ was used as a reference sample. N_2_ adsorption/desorption was determined by Brunauer-Emmett-Teller (BET) measurements using an ASAP-2010 surface area analyzer.

### 2.3. Adsorption Behavior of MB on the Synthesized Photocatalysts

To determine the adsorption behavior of MB on the synthesized photocatalysts, the typical preparation of the suspensions was as follows. Three given weights (0.200 g) of different photocatalysts were mixed in three aliquots of MB solutions (100 mL) with the same initial concentrations (5 × 10^−5^ mol/L), respectively. The suspensions were kept overweight in the dark and filtered after being centrifuged. The absorbance of the filtrated was then measured at the maximum band of 464 nm to determine the concentration of MB. The extent of equilibrium adsorption was determined from the decrease in the MB concentration detected after filtration. The Brunauer-Emmett-Teller (BET) surface area was estimated by using N_2_ adsorption data on a Micromeritics Tristar 3020 apparatus.

### 2.4. Evaluation of Photocatalytic Activity

All photocatalytic experiments were carried out under similar conditions in summer months of July and August. The sky is clear, and the sunrays are very intense in this period in the city of Urumqi (latitude: 43.46 N; longitude: 87.36 E), Xinjiang (China). All the solar experiments were performed on bright sunny days in order to avail maximum sunshine.

The photocatalytic activity of mesoporous BiVO_4_ nanorods was evaluated by photocatalytic degradation of MB aqueous solution. All photocatalytic reactions were performed under sunlight irradiation in a quartz beaker. The as-prepared photocatalyst (0.200 g) was dispersed in 100 mL of MB solution with initial concentration of 5 × 10^−5^ mol/L by stirring with a magnetic stirrer. The solution was stirred for 30 min in dark to allow the system to reach an adsorption/desorption equilibrium, then after a predetermined time, 5 mL of the solution was drawn and centrifugated. The optical absorption spectrum for the supernatant solution was recorded using a double-beam spectrophotometer (UV-2450, Japan and absorption at *λ*_max⁡_ = 664 nm for MB). The decolorization rate of MB was calculated by the following equation [27, 28]:

(1) $$D=\frac{C_{0}-C}{C_{0}}\times 100\text{\%}=\frac{A_{0}-A}{A_{0}}\times 100\text{\%},$$

where *C*_0_ and *A*_0_ were the concentration and absorbency of MB solution at maximum absorption wavelength of the initial solution and *C* and *A* were the concentration and absorbency of MB solution at maximum absorption wavelength after sunlight irradiation at any time.

For comparison, a 250 W medium pressure mercury arc lamp with a main wavelength of 450 nm_,_ was also used in the experiments to replace natural sunlight to investigate the photocatalytic properties of all the products in the laboratory in a similar quartz beaker.

## 3. Results and Discussion

### 3.1. Physicochemical Characterization of the As-Prepared BiVO_4_ Sample

**Figure 2:**
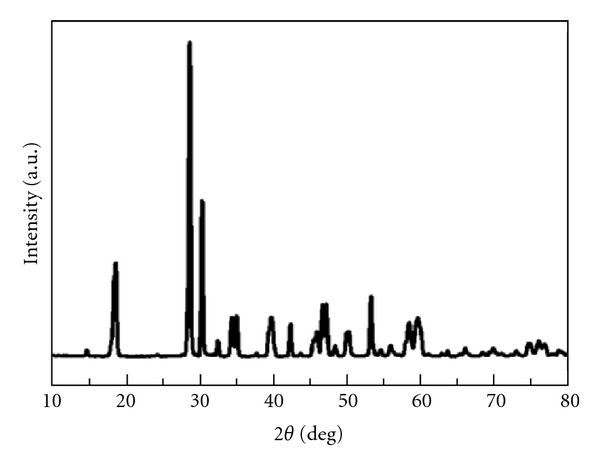
XRD pattern of the as-prepared mesoporous BiVO_4_ nanorods.

The phase and composition of the as-prepared product were investigated by using XRD measurement.  Figure 2 shows the XRD pattern of BiVO_4_ powder sample prepared by hydrothermal method at 160°C for 5 h. All diffraction peaks can be indexed to be a monoclinic phase BiVO_4_ (JCPDS no. 83–1699), and no peaks for any other phases or impurities are detected.

**Figure 3:**
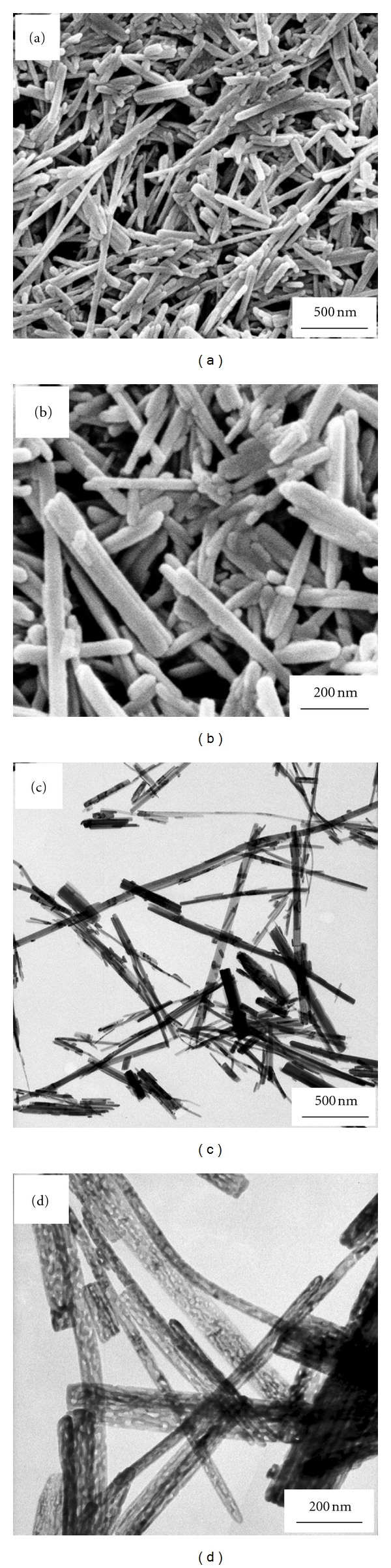
SEM (a, b) and TEM (c, d) images of the mesoporous BiVO_4_ nanorods with different magnifications.

 The morphology and microstructure of the as-prepared BiVO_4_ were revealed by SEM and TEM, as shown in Figure 3. The SEM images (Figures 3(a) and 3(b)) clearly demonstrate that the as-prepared products are almost entirely rods with length of 0.5–2 *μ*m and diameter of 50–80 nm. The rod-like morphology of the products is further confirmed by TEM images (Figure 3(c)), and the close-up observation of the samples revealed by the high-magnification TEM image (Figure 3(d)) shows that the rod possesses a porous interior structure, which increases the SSA of these nanorods and their photocatalytic reaction. Up to now, BiVO_4_ nanorods have been prepared by many groups [29–31], while BiVO_4_ nanorods with pores were seldom synthesized. During the reaction process, ethylenediamine plays an important role, and it not only adjusts the pH value of the mixed solution, but also directs the assembly of nanoparticles. A possible formation mechanism for mesoporous BiVO_4_ nanorods was tentatively illustrated. At first, the precursor formed by the reaction between BiO^+^ and VO_3_^−^ ions when the pH value of the mixed nitric acid solutions of Bi(NO_3_)_3_·5H_2_O and NH_4_VO_3_ was adjusted to 6-7 by ethylenediamine. When the precursor solution was treated under the hydrothermal condition at 160°C, the nanoparticles rearrange orderly to obtain rod-like structure under the direct of ethylenediamine. As for the formation of pores, it is probably attributed to the noncompactly accumulated nanoparticles.

 The SSA of all photocatalysts in this research also has been listed in Table 1. As shown in Table 1, SSR-BiVO_4_ displays a much lower SSA of 0.748 m^2^/g, while the as-prepared mesoporous BiVO_4_ nanorods exhibit a significantly higher SSA of 10.67 m^2^/g, *ca. *14 times higher than SSR-BiVO_4_. For commercial TiO_2_ (Degussa P25), its SSA is 50 m^2^/g [28]. The larger exposed surfaces of the mesoporous BiVO_4_ nanorods will serve as centers of condensing MB through a physical adsorption process, and the condensed MB would be degraded by generated hydroxyl radical [12].

**Figure 4:**
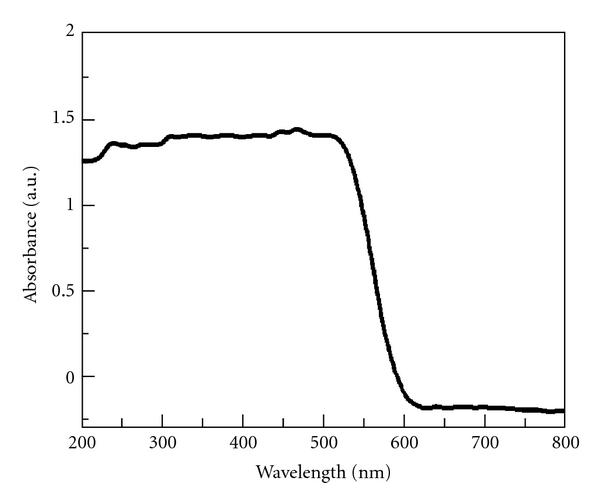
UV-Vis diffuse reflectance spectrum of the mesoporous BiVO_4_ nanorods.

 In general, the optical absorption property and migration of the light-induced electrons and holes of a semiconductor are recognized as the key factors in determining its photocatalytic activity [32]; thus it is the ideal way guiding the synthesis of semiconductors with good optical absorption property.  Figure 4 shows the room temperature UV-Vis diffuse reflectance spectrum of mesoporous BiVO_4_ nanorods with steep shape, indicating that the visible light absorption is ascribed to the band gap transition [33]. The mesoporous BiVO_4_ nanorods show strong photo-absorption property not only in the UV region but also in the visible light region until 600 nm. With respect to the wavelengths of absorption edges, they are determined by extrapolating the horizontal and sharply rising portions of the curves and defining the edge as the wavelength of the intersection [34]. The energy of the band gap of BiVO_4_ can be calculated as *ca.* 2.18 eV from its band gap absorption edge, which means that the as-prepared BiVO_4_ sample has a suitable band gap for photocatalytic decomposition of organic contaminants under sunlight irradiation.

### 3.2. Photocatalytic Activity for Degradation of MB in Water

#### 3.2.1. Adsorption Performance of MB on the Photocatalysts

A preliminary test with regard to the adsorption performance of MB on the catalysts is performed to obtain a few sets of stand control data, and the test is performed in the dark and the corresponding results are listed in Table 2. Note that the degree of MB adsorption on the photocatalysts has relatively direct relationship with their SSA in the absence of irradiation except for P25. The larger the SSA is, the stronger the adsorption property is. As for P25, its SSA is the biggest among the three photocatalysts, while its adsorption rate is the smallest, which may be attributed to the rich hydroxyl groups on the surface of TiO_2_, and many TiO_2_ nanoparticles seriously aggregated together due to hydrogen bond and thus resulted in its adsorption rate decreasing toward MB [8, 35, 36]. Besides the contribution from the effect of photocatalytic degradation, the total removal efficiency also comes from the assistance of the rapid attainment of adsorption equilibrium of the dye onto the photocatalysts. However, total removal of pollutant is enhanced dramatically by carrying out the photocatalytic degradation experiment under illumination of light. Especially, for the as-prepared mesoporous BiVO_4_ sample, its total removal for pollutant can be attributed to more than 71.2% of photodegradation capability in addition to the above-mentioned surface adsorption effect.

#### 3.2.2. Photocatalytic Activity for Degradation of MB in Natural Sunlight

**Figure 5:**
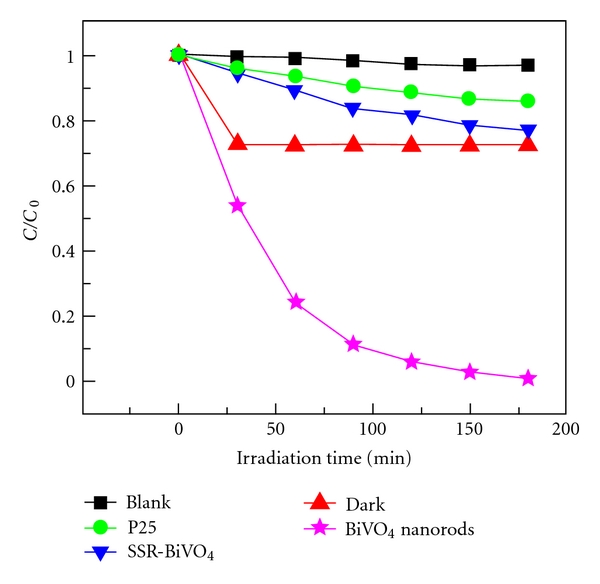
Photodegradation efficiencies of the MB as a function of irradiation time for different photocatalysts.

The photocatalytic activities of the photocatalysts are evaluated by photocatalytic degradation of MB aqueous solution under the natural sunlight irradiation through monitoring the intensity of the characteristic absorption peak at 664 nm of MB. For comparison, the photocatalytic performance of P25, SSR-BiVO_4_, and the direct photolysis of MB without catalysis (sunlight only) are also investigated under the same experimental conditions. The removal efficiency of MB under different conditions is reported in Figure 5. It can be seen from Figure 5 that the photolysis of MB is extremely slow without a photocatalyst under sunlight illumination, which is in consistent with the reference [37]. The decoloration rate of MB solution in presence of P25 is higher than that of direct photolysis, while it is only 14.2% over a period of 180 min, indicating that P25 is inactive under sunlight irradiation. The decrease of MB concentration in the presence of SSR-BiVO_4_ under sunlight is still small, only *ca.* 23.1% of MB was degraded in 180 min, which is attributed to the poor adsorption performance and difficult migration of electron-hole pairs of SSR-BiVO_4_. However, the decoloration rate of MB increases significantly by substituting SSR-BiVO_4_ for the mesoporous BiVO_4_ nanorods, and it reached 98.8% in 180 min. The as-synthesized BiVO_4_ nanorods are endowed with relatively higher SSA due to their 1D mesoporous rod-like structure, which provides richer active sites for MB adsorption and hence facilitates the enhancement in photocatalytic performance [38]. Nevertheless, the decolorizing of MB by the mesoporous BiVO_4_ nanorods is mainly attributed to the photocatalytic degradation rather than the simple adsorption.

**Figure 6:**
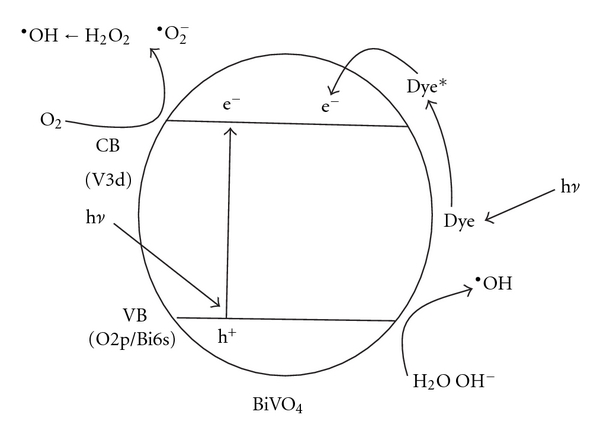
Photocatalytic mechanism for the mesoporous BiVO_4_ nanorods over MB.

To obtain some information about photocatalytic degradation mechanism of MB by the mesoporous BiVO_4_ nanorods, a possible pathway of the photoelectrons transfer excited by sunlight was tentatively introduced. As is known to all, monoclinic BiVO_4_ has a suitable band structure to absorb visible light due to its hybrid VB formed by Bi_6s_ and O_2p_ [18]. Based on the UV-Vis reflectance spectrum (Figure 4), the band gap of the mesoporous BiVO_4_ nanorods is calculated as 2.18 eV. Under the sunlight illumination, BiVO_4_ nanorods absorb the efficient photons (h*ν* ≥ 2.18 eV), and the electrons in the VB of BiVO_4_ are excited to CB, and thus the VB of BiVO_4_ is rendered the corresponding holes (Figure 6). Simultaneously, the adsorbed dye is excited to singlet or triplet states (dye*) due to the absorbing properties of dye for light, even if the wavelength of the light is longer than 600 nm [16], and then the electrons coming from the dye are injected into the CB of BiVO_4_. Additionally, the mesoporous structure of BiVO_4_ nanorods is beneficial to the migration of the photogenerated carriers; thus the probability of electron-hole recombination was reduced. The electrons in the VB of BiVO_4_ nanorods and the holes in the CB can participate in some reactions (as shown in (2)–(8)) assisted by the O_2_ dissolved in water and H_2_O, respectively, to generate many ^·^OH (Figure 6), which has strong oxidizing ability and is available to oxidize many organic pollutants [39, 40].

(2) $$\begin{array}{c} {\text{H}}_{2}\text{O}\to {\text{H}}^{+}+\text{O}{\text{H}}^{-} \end{array}$$

(3) $$\begin{array}{c} {\text{h}}^{+}+\text{O}{\text{H}}^{-}{\to \;}^{\cdot }{\text{OH}}^{-} \end{array}$$

(4) $$\begin{array}{c} {\text{e}}^{-}+{\text{O}}_{2}\to {\;}^{\cdot }{{\text{O}}_{2}}^{-} \end{array}$$

(5) $$\begin{array}{c} {\;}^{\cdot }{{\text{O}}_{2}}^{-}+{\text{H}}^{+}\to \text{H}{\text{O}}_{2}^{\cdot } \end{array}$$

(6) $$\begin{array}{c} 2\text{H}{\text{O}}_{2}^{\cdot }\to {\text{H}}_{2}{\text{O}}_{2}+{\text{O}}_{2} \end{array}$$

(7) $$\begin{array}{c} {\text{H}}_{2}{\text{O}}_{2}+{{\text{O}}_{2}}^{-}\to {\;}^{\cdot }\text{OH}+\text{O}{\text{H}}^{-}+{\text{O}}_{2} \end{array}$$

(8) $$\begin{array}{c} {\text{H}}_{2}{\text{O}}_{2}+\text{h}\upsilon \to 2^{\cdot }\text{OH} \end{array}$$

To quantitatively understand the reaction kinetics of the MB degradation in our experiments, the Langmuir-Hinshelwood model expressed by (9) was applied. When the pollutant is in the millimolar concentration range, (9) is well established for photocatalysis experiments [41, 42]:

(9) $$-\ln\frac{C}{C_{0}}=kt,$$

where *C*_0_, *C*, and *k* indicate the concentrations of dye in solution at time 0 and *t* and the apparent first-order rate constant, respectively. The reaction rate constant (*k*) can be determined *via* the first-order linear fit. Efficient photocatalysts usually display high values of *k*, and the typical values of *k* for the photocatalysts under sunlight irradiation are summarized in Table 3. The results demonstrate that the photocatalytic activity of mesoporous BiVO_4_ nanorods is much higher than those of SSR-BiVO_4_ and P25, indicating its preferable photocatalytic performance. 

**Figure 7:**
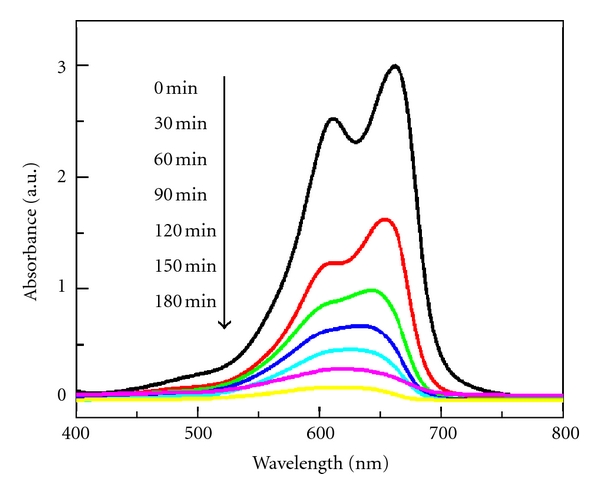
Temporal spectral changes of the MB in BiVO_4_ nanorods solution under sunlight irradiation; experimental conditions are otherwise identical to those of sunlight-irradiation BiVO_4_ dispersion in Figure 5.

In order to confirm that the MB solution was not simply decolorized during the degradation process, the absorption spectra of MB solutions as a function of irradiation time were further investigated (Figure 7). As seen in Figure 7, about 98.8% of MB in BiVO_4_ nanorods slurry is degraded after being irradiated for 180 min, and the spectral maximum shifted from 664 to 602 nm. As is known to all, the peaks between 600 and 700 nm are assigned to the absorption of the conjugated *π*-system [43, 44], and the absorption band in this region is chosen to monitor the temporal concentration changes of MB in BiVO_4_ suspensions. Because of the existence of weak electron-donor substituents, methyl groups, MB is vulnerable to be attacked by electrophilic species (^·^OH or h^+^) in the demethylation process, which is an important step in photocatalytic degradation process of MB [42]. When all or parts of the auxochromic groups (methyl or methylamine) degrade, the color of MB solutions becomes less intense, indicating that MB is N-demethylated in a stepwise manner, which is confirmed by the gradual peak wavelength shifting toward the blue region, as shown in Figure 7.

#### 3.2.3. Comparison of Photocatalytic Degradation of MB Solutions with Different Photocatalysts under Different Light Irradiation

The ultimate goal of the study by using the mesoporous BiVO_4_ nanorods as photocatalyst waste water treatment is to apply this technology utilizing solar energy as the energy source. Accordingly, this work addresses the direct comparison of artificial and natural energy sources in order to apply this technique in the wastewater treatment plant. The comparison experiments were carried out by using other two different photocatalysts, SSR-BiVO_4_ and P25.

Different light sources have an important effect on the photocatalytic properties of the photocatalysts. In order to further confirm that sun will be a promising and ideal light source, a 250 W medium pressure mercury arc lamp, whose main wavelength is 450 nm, was also used in the experiment to replace natural sunlight to investigate the photocatalytic properties of all products in the laboratory in a similar quartz beaker. Table 3 shows the comparative results of the photocatalytic properties of different photcatalysts under different light sources irradiation (5 × 10^−5^ mol/L dye concentration, 90 min light irradiation). It could be seen from Table 1 that all photocatalysts showed higher photocatalytic properties under sunlight irradiation than artificial light, for which the reason could be attributed to the fact that the artificial light has the main wavelength of 450 nm while sunlight has partial UV light besides visible light. Thus it can be seen that sun will be a promising light source in the degradation process of dye. Moreover, it is both applicable and cost-saving to use sun as light source.

To confirm the stability of the high photocatalytic activity of the mesoporous BiVO_4_ nanorods, the photocatalytic degradation performance of MB over the circulated mesoporous BiVO_4_ nanorods under sunlight irradiation was investigated (Table 4). After five recycles for the photocatalytic degradation of MB, the photocatalyst still exhibits high photocatalytic activity, which reveals that the as-prepared BiVO_4_ nanorods have high stability and do not photocorrode during the photocatalytic oxidation of the model pollutant MB. The high photocatalytic activity and stability of the mesoporous BiVO_4_ nanorods are especially beneficial to its practical application.

## 4. Conclusions

In conclusion, we first synthesized sunlight-driven mesoporous BiVO_4_ nanorods *via* a simple hydrothermal method. The as-prepared monoclinic BiVO_4_ photocatalyst exhibited high specific surface area of 10.67 m^2^/g. The mesoporous BiVO_4_ nanorods presented strong absorptions in visible light region up to 660 nm as well as in the UV region. The band gap was estimated to be *ca.* 2.18 eV. The excellent photodegradation rate of as-prepared BiVO_4_ nanorods was up to 98.8% in 180 min under natural sunlight irradiation, much higher than the SSA-BiVO_4_ (*ca.* 8%) and the commercial P25 (*ca.* 6%) under the same conditions, owing to the higher specific surface area and the appropriate band gap. In addition, the hydrothermal route applied here is effective, convenient, low-cost, and environmentally friendly, and thus worth to be extended to other photocatalyst systems.

## Figures and Tables

**Table 1:** BET SSA and reaction rate constant *k* of mesoporous BiVO_4_, nanorods, SSR-BiVO_4_ and P25, respectively.

Sample	SSA_BET_ (m^2^/g)	*k* (min^−1^)
BiVO_4_ nanorods	10.67	0.0243
SSR-BiVO_4_	0.748	0.0015
P25	50	0.0009

**Table 2:** Preliminary test for the adsorption and degradation performances of MB on mesoporous BiVO_4_, nanorods, SSR-BiVO_4_ and P25, respectively.

	BiVO_4_ nanorods	SSR-BiVO_4_	P25
Initial concentration (10^−5^ mol/L)	5	5	5
Adsorption removal^a^ (10^−5^ mol/L/%)	1.380/27.6	0.570/11.4	0.490/9.8
Photodegradation removal^b^ (10^−5^ mol/L/%)	3.560/71.2	0.585/11.7	0.220/4.4
Total removal^c^ (10^−5^ mol/L/%)	4.940/98.8	1.155/23.1	0.710/14.2

^a^Total removal in the dark using photocatalyst.

^b^Total removal−adsorption removal = photodegradation removal.

^c^Total removal under light illumination using photocatalyst.

**Table 3:** Effect of light sources upon the photocatalytic properties of different photocatalysts (5 × 10^−5^ mol/L dye concentration, 90 min light irradiation).

Light sources	BiVO_4_ nanorods	SSR-BiVO_4_	P25
Sun light (%)	99.8	23.1	14.2
Artificial light (%)	81.2	17.3	9.8

**Table 4:** Degradation rates of the MB over the mesoporous BiVO_4_ nanorods.

	Raw solution	1st use	2nd use	3rd use	4th use	5th use
Absorbance (a.u.)	2.981	0.006	0.012	0.009	0.009	0.012
Degradation ratio (%)		99.8	99.6	99.7	99.7	99.6
